# Is it advisable for Asians to drink milk, especially those at risk of osteoporosis?

**DOI:** 10.3389/fnut.2025.1586623

**Published:** 2025-06-25

**Authors:** Hao-tian Jiao, Yu-Shan Yue, Shuai Yuan, Xiao-jie Zhou, Chong Li

**Affiliations:** ^1^Department of Orthopedics, The First People’s Hospital of Kunshan, Gusu School, Nanjing Medical University, Suzhou, Jiangsu, China; ^2^Kunshan Biomedical Big Data Innovation Application Laboratory, Suzhou, Jiangsu, China; ^3^Department of Rehabilitation, Affiliated Kunshan Hospital of Jiangsu University, Suzhou, Jiangsu, China; ^4^Department of Orthopedics, Affiliated Kunshan Hospital of Jiangsu University, Suzhou, Jiangsu, China

**Keywords:** osteoporosis, bone health, milk allergy, calcium, lactose intolerance, milk

## Abstract

Osteoporosis poses a significant health challenge globally, with particularly high rates anticipated in the Asian region in the forthcoming years. This region has been identified as a potential hotspot for increasing incidences of osteoporosis. Unfortunately, the Asian population has a low consumption of milk and dairy products, which may exacerbate this situation. A deficiency of calcium ions and inadequate consumption of milk and other dairy products significantly exacerbate the risk of developing osteoporosis. Milk, which is a rich source of protein, vitamin D, and various essential minerals, stands out as an optimal dietary choice for managing and potentially mitigating osteoporosis in individuals of Asian descent. Consuming milk provides these vital nutrients while playing a crucial role in maintaining bone health and preventing the loss of bone density. Here, we delve into the multiple nutritional benefits of milk for individuals susceptible to osteoporosis, and discuss comprehensive strategies to overcome challenges that are prevalent within the Asian demographic, such as milk allergies and lactose intolerance.

## 1 Introduction

Osteoporosis is a systemic metabolic bone disorder characterized by a reduction in bone mineral density (BMD) and the deterioration of bone microarchitecture (1, 2). The International Osteoporosis Foundation estimates that more than 200 million individuals worldwide are affected by osteoporosis (3), highlighting the significance of osteoporosis as a global public health issue with growing importance in Asian countries (4, 5). Epidemiological studies indicate that osteoporosis affects approximately 20–30% of older adults in major Asian countries such as China and India, with postmenopausal women facing particularly heightened risk due to estrogen deficiency and age-related bone loss (6). Due to the rapidly aging population in Asia, the number of hip fracture is expected to increase substantially between 2018 and 2050 based on predicted changes, projections indicate that by 2050, over half of all hip fractures globally will take place in this region (7, 8). Models of coordinated post-hip fracture care such as FLS are being established in parts of Asia to reduce secondary fracture risks and mortality (9). Many countries and regions in Asia have revised their clinical guidelines for osteoporosis prevention and treatment. These updates focus on lowering the risk of osteoporotic fractures and enhancing the quality of care for patients with osteoporosis. The revised guidelines incorporate the most recent research findings and offer practical advice for healthcare providers to effectively prevent, diagnose, and manage osteoporosis in clinical practice (10–12).

**Figure 1:**
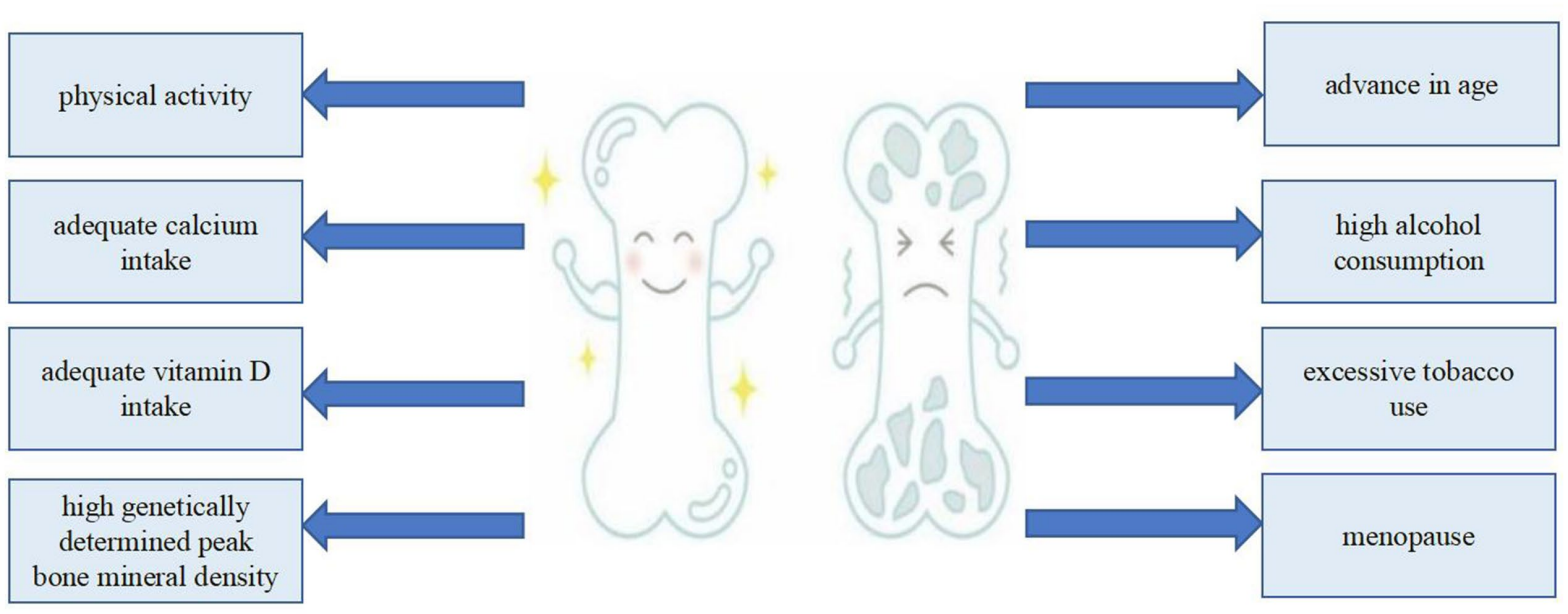
Protective and harmful factors of osteoporosis.

Numerous factors are involved in the development of osteoporosis, including genetically determined peak BMD, age, menopause, inadequate calcium or vitamin D intake, physical inactivity, excessive tobacco use, high alcohol consumption, and excessive caffeine intake (13) (Figure 1). An invaluable dietary staple for thousands of years, milk has played a critical role in human nutrition (14, 15). Milk, along with its derivative products, is celebrated for its extensive nutritional advantages, which have been substantiated through numerous scientific studies conducted over many decades. As a result, milk and its derivatives are acknowledged not only for their essential nutrients, but also for their indispensable role in meeting the dietary needs of the vast majority of people across the globe. This widespread recognition underscores the role of milk as a foundational food item in diverse cultures and societies (16, 17). Each liter of milk (Table 1) contains 1,180 mg of calcium (18), which is of great significance to the prevention and treatment of osteoporosis (19, 20). However, drinking milk is not a traditional part of the culture in many Asian countries, resulting in significantly lower consumption compared with that in European countries (21).

**Table 1:** Nutrient content of milk (14, 32, 43, 143).

Ingredient name	Concentration/Content (Unit)
Water (%)	87.6
Lactose (g/L)	44–66
Protein (g/L)	30–39
Fat (g/L)	33–54
Total casein (g/L)	24.6–28.0
Calcium Content (mg/L)	1,180
Vitamin D (μg/L)	0.3

Meanwhile, Asian populations are at notable risk of calcium deficiency because of their dietary habits and nutritional intake, which often lead to inadequate levels of this essential mineral crucial for optimal bone health (22) (Table 2). Lactose intolerance is the most common form of food intolerance globally, affecting a significant portion of the population across various regions and cultures (23). Lactose intolerance affects approximately 70% of the adult population worldwide, with the highest prevalence in Eastern Asia, where > 90% of the inhabitants are classified as lactose intolerant.

**Table 2:** Average milk consumption and calcium intake in different Asian countries.

Author, year	Journal	Region	Survey year	Milk consumption	Calcium intake
Yuna He (144), 2016	Proceedings of the Nutrition Society	China	2013	8.7 kg/year	366 mg/d
Chittari Venkata Harinarayan (31), 2021	Frontiers in Endocrinology	India	2011	≤103 mL/d	≤370 mg/d
Shuhua Yang (145), 2023	BMC Nutrition	China	2011	35 g/day	–
Xiaona Na (25), 2022	Nutrients	China	2012	26.47 g/day	–
Aki Saito (146), 2019	Nutrients	Japan	2016	63 g/day	–
E M Balk (30), 2017	Osteoporosis International	Thailand	2008	–	313 mg/d
		South Korea	2012	–	483 mg/d
		Pakistani	2008	–	462 mg/d
		Malaysia	2003	–	399 mg/d
Hiroaki Ohta (22), 2016	Osteoporosis and Sarcopenia	Japan	2014	–	<500 mg/d

In light of the relevant cultural factors, dietary practices, and high prevalence of lactose intolerance, there remains a considerable ongoing debate regarding the suitability of milk consumption for Asian individuals, particularly those at increased risk for osteoporosis. This review aims to provide a comprehensive examination of the literature, with a focus on the complex relationships between milk and osteoporosis, particularly in high-risk Asians, while also offering an objective overview of the most significant findings on the topic.

## 2 Method

This review comprehensively evaluates whether Asian populations, especially those with osteoporosis, are suitable for drinking milk, despite people may have some concerns.

The search strategy was conducted exclusively in the PubMed database, using keywords including “osteoporosis,” “bone mineral density,” “milk allergy,” “lactose intolerance.” When searching for articles related to average milk consumption, calcium, and vitamin intake among Asian populations, we employed a systematic literature retrieval approach as follows: ((“Calcium”[MeSH] OR calcium) AND (intake OR consumption OR “dietary intake”)) AND ((“Milk”[MeSH] OR milk OR “Dairy Products”[MeSH] OR dairy OR “milk consumption” OR “dairy consumption”)) AND ((“Vitamin D”[MeSH] OR “vitamin D” OR cholecalciferol OR ergocalciferol) AND (intake OR consumption OR “dietary intake”)) AND ((“Asia”[MeSH] OR Asia OR Asian OR “Asian populations”)) AND (Humans[MeSH]). When searching for articles on the relationship between milk consumption and bone density among Asian populations, we employed the following search strategy: ((Asia[tiab] OR Asian[tiab] OR “East Asia”[tiab] OR “Southeast Asia”[tiab] OR China[tiab] OR Japan[tiab] OR Korea[tiab] OR India[tiab]) AND (“bone density”[tiab] OR “bone mineral density”[tiab] OR BMD[tiab] OR “bone mass”[tiab]) AND (milk[tiab] OR “cow’s milk”[tiab] OR “fortified milk”[tiab])). The screening process was conducted independently by three researchers (Yu-Shan Yue, Shuai Yuan, Xiao-jie Zhou), each systematically reviewing the titles, abstracts, and full texts of identified articles to extract key study characteristics, methodologies, and primary outcomes. Any discrepancies or disagreements encountered during study selection or data extraction were resolved through detailed discussion; if consensus could not be reached, a formal vote was conducted by the graduate research team to determine inclusion. In cases where the voting results were inconclusive or closely divided, a senior researcher (Chong Li) conducted a detailed evaluation of the article titles and abstracts, applying predefined inclusion criteria to determine the article’s relevance and suitability for the research topic. This additional step ensured that the final selection aligned closely with the study objectives and maintained methodological rigor. A standardized quality assessment of all included studies was performed using established evaluation tools to ensure methodological rigor and the reliability of findings. The literature search encompassed publications from January 1, 2004, to December 31, 2024.

To obtain comprehensive and accurate information on nutrition recommendations across Asian countries, we conducted a systematic review of official dietary guidelines and policy documents published by governmental and international authorities. Specifically, we identified and extracted data from the most recent editions of the Chinese Dietary Guidelines, the Japanese Dietary Reference Intakes, the Indian Council of Medical Research (ICMR) recommendations, and the National Institute of Nutrition guidelines for Vietnam. For Saudi Arabia, we consulted official documentation provided by the Saudi Food & Drug Authority (SFDA). To supplement these national guidelines and ensure consistency with international standards, we also reviewed relevant publications and recommendations from the Food and Agriculture Organization (FAO) and the World Health Organization (WHO). All sources were accessed through official government and institutional websites to ensure the authenticity and reliability of the information. Data extraction was performed independently by two reviewers (Shuai Yuan, Xiao-jie Zhou), with any discrepancies resolved through discussion or consultation with a third reviewer (Yu-Shan Yue).

**Figure 2:**
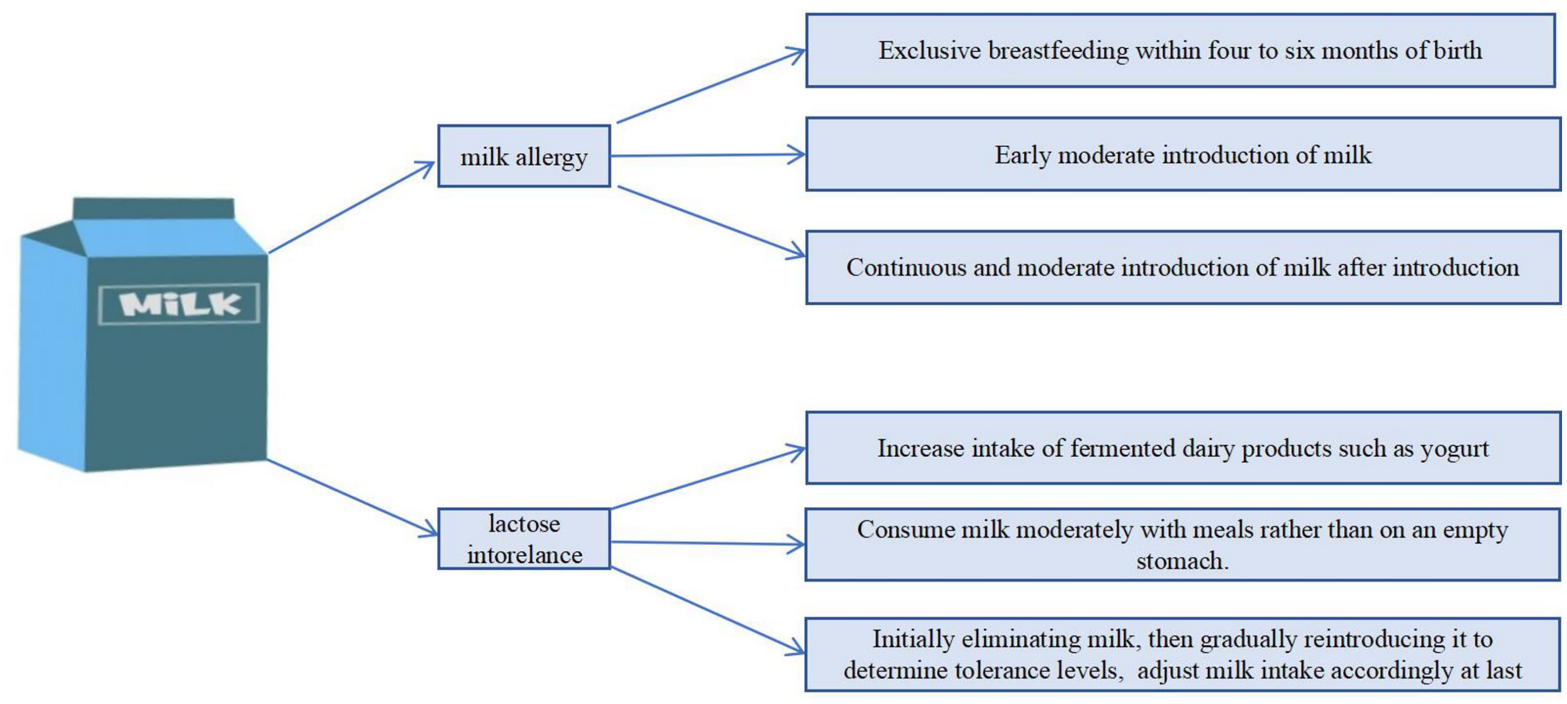
Potential approaches for managing milk allergy and reducing lactose intolerance.

The review applied a qualitative synthesis approach, integrating findings from diverse study designs to explore the impact of dairy product consumption and key nutrient intakes on bone health in Asian populations. Challenges related to lactose intolerance and milk allergy were also examined to provide evidence-based dietary recommendations (Figure 2).

## 3 Low milk consumption and deficient calcium and vitamin D intake in Asian populations

In many Asian cultures, dairy products have historically not been included in traditional diets (24, 25).

At the same time, the average milk consumption and calcium ion intake in these countries have not reached the recommended standards (Table 2). According to the latest data (2022) from the International Food and Agriculture Organization (FAO), the average annual per capita milk consumption in Asia is 60.42 kg, with substantial regional variation. In Southeast Asia, countries such as Thailand, Myanmar, and Vietnam report relatively low intakes at 28.82 kg, 8.15 kg, and 37.21 kg per year, respectively. In East Asia, average consumption is 31.72 kg in China, 62.15 kg in Japan, and 31.35 kg in South Korea. In contrast, South Asian countries such as India and Pakistan have notably higher levels of milk consumption, with 81.8 kg and 114.31 kg per year, respectively. Central Asian countries report the highest consumption, with Mongolia at 240.83 kg and Kazakhstan at 283.76 kg per year. In West Asia, per capita milk intake also varies, with Israel at 180.97 kg, Saudi Arabia at 62.67 kg, and Lebanon at 114.57 kg annually.

Some Asian countries have less developed sales and production of milk due to incomplete production and supply chains compared with Western counterparts, contributing to a limited supply that makes milk less common in the daily diet. Additionally, economic constraints arising from the significant gap in economic development between Asia and the West have also played a role in this disparity in areas where dairy farming is uncommon, milk may be relatively expensive, making it difficult for ordinary consumers to obtain milk. Fortunately, many developing countries, particularly in East Asia and Southeast Asia, have seen significant increases in the consumption of dairy products, driven by economic development and heightened awareness of health and nutrition over the past few decades (26, 27). Although milk intake and production have risen substantially in Asia, the levels remain considerably lower than those observed in Western countries (28, 29). Research has shown that the calcium intake of Asian populations is significantly lower than the recommended standard, with an average calcium intake of approximately 500 milligrams per day or lower in Japan and South Korea (22), and is notably below that observed in Western populations. In various nations across South, East, and Southeast Asia, daily calcium intake often falls below 400 mg (Table 2). According to the systematic review “Global dietary calcium intake among adults: a systematic review,” a comprehensive search was conducted across 13 electronic databases and through expert consultation to identify the most representative national dietary calcium intake data among adult populations worldwide. Studies were screened in a double-blind manner to ensure suitability and minimize bias. The findings indicate that in many Asian countries, the average dietary calcium intake remains below 500 mg per day. For example, the reported mean daily calcium intakes are 313 mg in Thailand, 483 mg in South Korea, 462 mg in Pakistan, and 399 mg in Malaysia, all of which fall significantly short of international recommendations for calcium intake (30). Moreover, according to the review “Recent Nutritional Trends of Calcium and Vitamin D in East Asia,” recent national nutrition surveys in Japan indicate a gradual decline in calcium intake to approximately 480 mg per day. Additionally, an increasing proportion of individuals with low vitamin D levels has been observed. The authors recommend that calcium intake should be increased among Asian populations to address these nutritional concerns (22). Another review shows that although India is the largest producer of milk and grains, the primary source of dietary calcium in the population is non-dairy foods. Studies have shown that grain consumption is highest and milk and dairy product intake is lowest among rural and tribal populations, whereas urban and rural populations exhibit similar patterns of grain, milk, and dairy consumption. One contributing factor to the generally low calcium intake (<370 mg/day) in India is the relatively small proportion of calcium derived from dairy sources (31). Current evidence indicates that calcium intake across many Asian countries is substantially below recommended levels, with most populations consuming less than 500 mg per day. This inadequate intake is influenced by dietary patterns that rely more on non-dairy sources. The declining trend in calcium intake, combined with increasing rates of vitamin D deficiency, highlights an urgent need for targeted nutritional strategies to address these micronutrient gaps in Asia.

Adequate calcium intake is necessary throughout a person’s life (Table 3) (32). One possible explanation for this phenomenon is that many Asian diets traditionally focus on cereals, legumes, and vegetables, with less emphasis on dairy products, which are a major source of calcium in Western diets. Although some plant-based foods like certain leafy greens, beans, and tofu provide calcium, they are often eaten in amounts that do not adequately fulfill the calcium intake recommendations set by health organizations. Given the economic challenges that limit the ability of some individuals to purchase supplements or calcium-fortified products, and the generally insufficient calcium intake among Asians (<500 mL in general), it is crucial to find other ways to increase their calcium intake.

**Table 3:** Calcium intake recommendations for various Asian countries (32).

Country	Region	Recommended calcium intake	Source of data
China	East Asia	800 mg/ day (adult)	the Chinese Dietary Guidelines
Japanese	East Asia	750 mg/ day (male) 650 mg/ day (female)	Dietary Reference Intakes for Japanese
India	South Asia	1,000 mg/ day (adult)	Indian Council of Medical Research (ICMR)
Kazakhstan	Central Asia	1,000 mg/ day (adult)	Food and Agriculture Organization (FAO) World Health Organization (WHO)
Vietnam	South-east Asia	1,000 mg/ day (adult)	National Institute of Nutrition (Vietnam)
Thailand	South-east Asia	800 mg/ day (adult)	Thai Food and Drug Administration (Thai FDA)
Saudi Arabia	Western Asia	1,000 mg/ day (adult)	Saudi Food & Drug Authority (SFDA)
Lebanon	Western Asia	1,000 mg/ day (adult)	Food and Agriculture Organization (FAO) World Health Organization (WHO)
International standards	–	1,000 mg/day (adult)	Food and Agriculture Organization (FAO) World Health Organization (WHO)

According to the United States Institute of Medicine (IOM), vitamin D status is classified as follows: deficiency is defined as a serum 25(OH)D concentration of less than 12 ng/mL (30 nmol/L); insufficiency is 12–20 ng/mL (30–50 nmol/L); sufficiency is at or above 20 ng/mL (50 nmol/L); and concentrations exceeding 50 ng/mL (125 nmol/L) are considered potentially excessive. At present, there is no completely unified international standard for the recommended intake of vitamin D, Several Asian countries have established recommendations for vitamin D intake. For instance, the Chinese Nutrition Society advises a daily intake of 10 μg (400 IU), a standard that is also adopted in South Korea. For individuals over 65 years of age, South Korea recommends an increased intake of 15 μg (600 IU) per day. In Japan, the recommended daily intake is 8.5 μg (340 IU). At the same time, many Asian countries have not established their own national standards for vitamin D intake and instead adopt the recommendations of IOM. According to these guidelines, the recommended daily intake for adults aged 19–70 years, as well as for pregnant and lactating women, is 15 μg (600 IU), while for individuals over 70 years of age, the recommended intake increases to 20 μg (800 IU) per day. There is a paucity of literature specifically addressing the average dietary intake of vitamin D in Asian countries. Nonetheless, some existing studies can provide insight into the prevailing vitamin D deficiency status within populations across this region. According to a survey of vitamin D in South Asian population, mean serum vitamin D concentrations ranged between 4.7 and 32 ng/mL, with a weighted mean of 19.15 ng/mL (SD 11.59 ng/mL). The highest deficiency rates were observed in Pakistan (73%; 95% CI: 63–83%), followed by Bangladesh (67%; 95% CI: 50–83%), India (67%; 95% CI: 61–73%), Nepal (57%; 95% CI: 53–60%), and Sri Lanka (48%; 95% CI: 41–55%). Notably, deficiency prevalence was higher among females than males across the region (33). Research conducted in northeast and northwest China revealed a high rate of vitamin D deficiency, with the average serum 25(OH)D level being notably low at approximately 30 nmol/L (7). According to another survey of vitamin D in Asian population, the mean serum 25-hydroxyvitamin D concentration was 49.39 nmol/L. Among participants, 20.93% exhibited levels below 25 nmol/L, 22.82% below 30 nmol/L, 57.69% below 50 nmol/L, and 76.85% below 75 nmol/L (34). Existing literature indicates that the prevalence of vitamin D deficiency is generally high in many Asian countries. Although direct data on dietary vitamin D intake are limited, multiple studies have shown that serum 25(OH)D levels tend to be low, with deficiency rates higher among women than men. Overall, the vitamin D status of Asian populations is concerning, underscoring the urgent need to strengthen vitamin D supplementation and related nutritional interventions to improve public health outcomes.

## 4 Nutrients provided by milk and milk-based derivatives

Milk comprises approximately 87% water, with 3.5% protein and roughly 5% lactose on average, and a fluctuating fat content that ranges from 0.5% in skimmed milk to between 3 and 4% in whole milk. Additionally, milk contains approximately 1.2% minerals, primarily calcium and phosphorus, which are crucial for human health (35). Milk protein is a significant component (36) of cow’s milk, which is distinctively characterized by a composition of 80% caseins and 20% whey proteins (37). Caseins and whey are considered nutritionally superior to plant-based proteins because of their complete profiles of essential amino acids and their high levels of digestibility, making them highly beneficial for nutrition. Lactose, which constitutes the primary carbohydrate in milk and its derivative dairy products, has been shown to enhance the absorption rates of critical trace minerals, including calcium, phosphorus, and magnesium. This functional role of lactose contributes significantly to the nutritional value of dairy (38, 39). Milk lipids are primarily present as milk fat globules suspended in the aqueous phase (40). These globules comprise a core of triacylglycerols and are enclosed by a membrane made up of phospholipids, glycolipids, proteins, and cholesterol. This milk fat globule membrane serves as an emulsifier, stabilizing the lipid droplets within the emulsion (41). Milk is a rich source of major minerals, such as calcium, magnesium, phosphorus, and potassium, along with the trace minerals iodine, selenium, and zinc (42). Overall, milk is a nutrient-dense food containing all three primary macronutrients and a wide array of essential vitamins and minerals (Table 1) (14, 32, 43). Relative to milk, yogurt contains a higher concentration of bioactive peptides, provides beneficial probiotic bacteria, and has a reduced lactose content (30–40 g/L). These properties make yogurt particularly suitable for populations with a high prevalence of lactose intolerance, such as those in many Asian countries, as the fermentation process significantly decreases lactose levels and the presence of live cultures can aid in lactose digestion. Furthermore, regular consumption of yogurt may contribute to better gastrointestinal tolerance and improved nutrient absorption in individuals who are sensitive to lactose (32, 44). In addition, fortified milk products are notable for their substantially higher levels of vitamins D and A when compared to regular milk (1.0–2.0 μg/100 mL), addressing common micronutrient deficiencies observed in several Asian populations. Some fortified dairy products also contain increased calcium concentrations, which can further support bone health, particularly in populations with traditionally low dairy intake (45, 46). Given their nutrient composition, these products have the potential to serve as beneficial foods for populations in Asia at risk of osteoporosis.

## 5 Roles of milk components (protein, minerals, and vitamins) in bone health

### 5.1 Impact of milk protein on bone health

The importance of protein to the human body cannot be overemphasized, and milk contains a high level of protein (47). Protein homeostasis is a key factor in maintaining cellular homeostasis, health, and disease (48). Protein consumption is essential for maintaining bone integrity, and inadequate protein intake has been linked with detrimental impacts on bone health (49). As a key component of bone structure, protein plays a pivotal role in bone health, significantly facilitating bone development during the crucial peripubertal phase while helping to prevent bone density loss in subsequent phases of life. This dual functionality underscores the importance of adequate protein intake in maintaining bone integrity over time (50–53).

Milk proteins are divided into caseins (80%) and whey proteins (20%) (54).

Whey proteins: accounting for about 20%. It mainly includes *β* -lactoglobulin, *α* -lactalbumin, serum albumin and immunoglobulins. Whey protein is easily absorbed, rich in essential amino acids, and has high bioavailability (55).

Caseins: A substantial body of research has demonstrated that caseins, comprising 80% of the protein content of milk, play a significant role in promoting the absorption of calcium and enhancing BMD. This evidence underscores the importance of caseins in supporting skeletal health (56). Caseins constitute a group of proteins primarily made up of α-caseins (including αs1 and αs2 caseins), along with *β*-caseins and *κ*-caseins (57). The phosphorylated serine-rich regions in α-caseins facilitate calcium binding and micelle formation but also present immunogenic epitopes triggering allergic responses in sensitive individuals. Studies have shown that αs1-casein, in particular, is strongly associated with eliciting immune reactions in children with cow’s milk allergy (58). β-casein is one of the main casein proteins in milk, with good emulsifying and nutritional properties. It can effectively form a micelle core and participate in stabilizing the casein micelle structure. In addition, β-casein contains some bioactive peptides that can exert physiological functions such as anti-hypertension and immune regulation after enzymatic hydrolysis. κ-casein is mainly localized on the surface of casein micelles, playing a role in protecting and stabilizing the micelle structure. κ-casein prevents excessive aggregation between micelles. In addition, kappa casein has certain antibacterial functions, which help protect milk fat and casein from microbial damage (59). With growing consumer recognition of the critical role of protein in maintaining health, milk is gaining prominence as a readily accessible source of protein (60). Traditional Asian diets are generally low in high-quality protein and dairy products. According to FAO data, while per capita protein intake in many West and East Asian countries has reached or exceeded recommended levels with economic development, it remains lower than in developed countries and is still insufficient in parts of Southeast and South Asia, especially in rural areas. Additionally, the proportion of animal-based protein in Asian diets is relatively low. Milk protein, with its high digestibility and absorption rate, can efficiently compensate for dietary deficiencies and improve bone metabolic health. Beyond directly supporting bone health, milk protein also helps maintain or increase muscle mass, thereby reducing the risk of sarcopenia and falls in the elderly, and indirectly lowering the incidence of fractures among populations at risk for osteoporosis (61).

### 5.2 Impact of milk minerals on bone health

Milk is recognized as a valuable dietary source of calcium, an essential nutrient crucial for the development and maintenance of healthy bones (18, 35, 62). Furthermore, calcium constitutes a primary element of bone tissue. Calcium intake that satisfies the recommended daily intake (Table 2) contributes to both the density and strength of the skeletal structure, helping to mitigate the degradation of bone mass typically seen with advancing age (63, 64). Asia is undergoing the most accelerated population aging worldwide, with countries like Japan already demonstrating a particularly high proportion of elderly citizens. In recent years, nations such as China, Thailand, and Vietnam have transitioned into aging societies, and their older populations are expected to grow substantially in the near future. Furthermore, countries including India and Indonesia are projected to face similar demographic shifts and associated challenges in the coming decades. The rapid increase in the elderly population across the region is accompanied by a heightened risk of osteoporosis (4, 65). In addition to constituting a significant structural element of bone, calcium ions are essential regulators of bone metabolism. Extracellular calcium is crucial for the regulation of bone metabolism (66), and calcium ions have been demonstrated to stimulate the activity of osteoblasts (67). Osteoblasts play a pivotal role in bone health by synthesizing thriche bone extracellular matrix, facilitating the deposition of calcium, and expressing various osteoclastogenic factors. These activities are essential for the formation and maintenance of bone structure, underscoring the integral function of osteoblasts in skeletal development and regeneration (64, 68). Additionally, low concentrations of calcium ions stimulate the release of parathyroid hormone (PTH) by the parathyroid gland (69, 70). While PTH promotes bone resorption and releases calcium ions into the bloodstream to supplement blood calcium levels, in the long run, it can lead to bone loss (71–73). In summary, insufficient dietary calcium is recognized as a significant risk factor for the development of osteoporosis (74); therefore, the rich calcium content in milk (1,180 mg/L) makes it a leading option for dietary calcium supplementation. In light of the rapidly aging population in Asia, the increasing urbanization that limits physical labor, and the prevalent insufficiency of dietary calcium intake, milk emerges as an essential dietary component. Owing to its rich calcium content (1,180 mg/L), ease of consumption, broad availability, and cost-effectiveness, milk plays a vital and irreplaceable role in mitigating the escalating risk of osteoporosis throughout the region.

Magnesium is the structural component of bones. Approximately 60% of magnesium in the body is stored in bone tissue, where it forms part of the bone matrix and hydroxyapatite crystals, playing a vital role in bone strength and resilience (75). Magnesium also regulates calcium metabolism and parathyroid hormone function. Magnesium is involved in the synthesis and activation of parathyroid hormone (PTH) and vitamin D, indirectly controlling calcium absorption in the intestine and its redistribution in bones. Magnesium deficiency impairs PTH secretion and affects bone metabolism (76). Last but not least, magnesium influences bone formation and mineralization. Adequate magnesium intake promotes osteoblast activity and new bone formation, whereas magnesium deficiency is associated with reduced bone density and an increased risk of fractures (77). For the general Asian adult population, the advised daily intake of magnesium typically falls within the range of 240 to 400 mg (78). A serving of milk can provide a valuable source of this essential nutrient (30 mg) for Asians at increased risk of osteoporosis.

### 5.3 Impact of vitamin D on bone health

**Figure 3:**
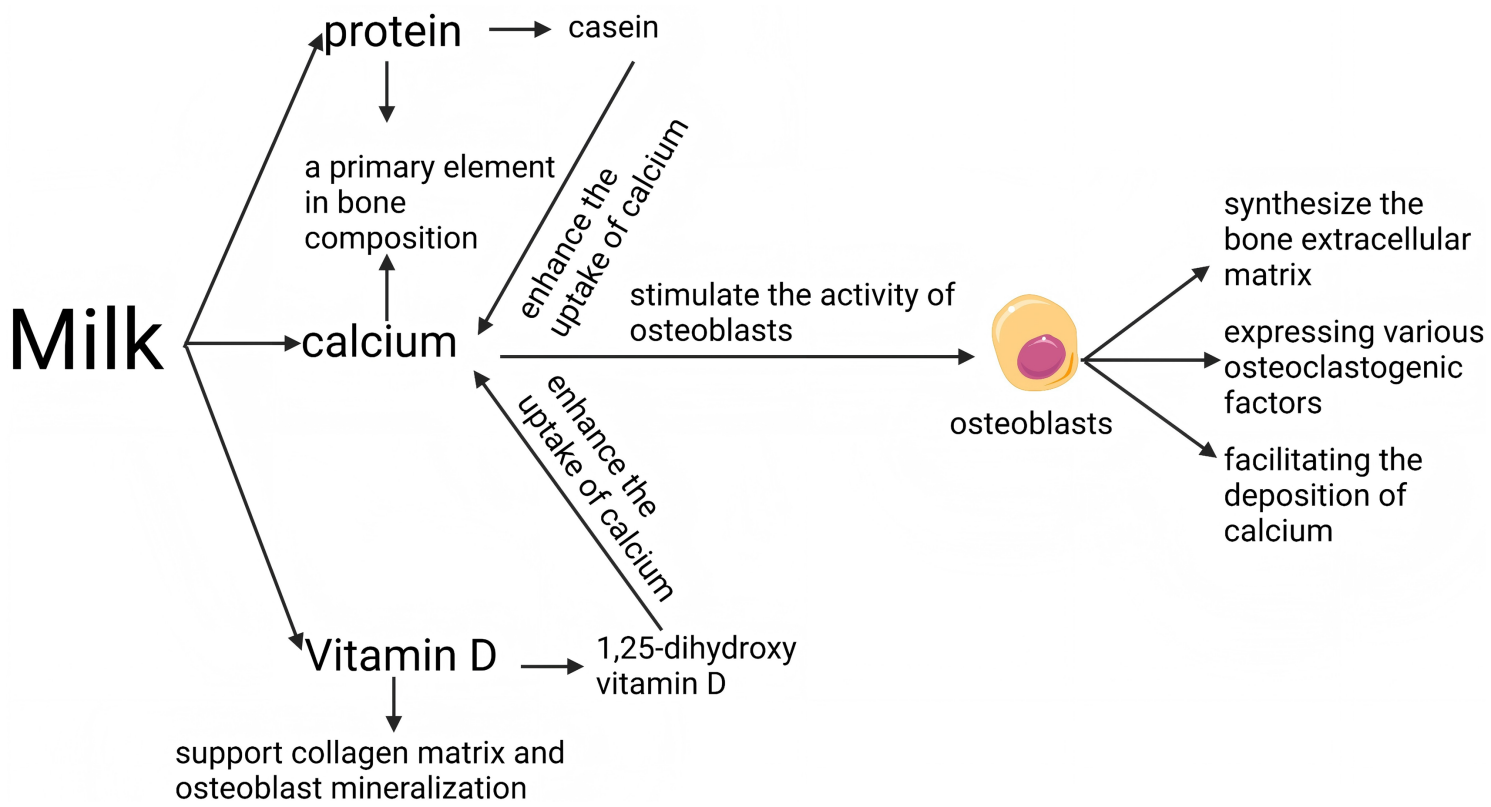
Effect of milk on osteoblasts.

Historically, Vitamin D has been utilized extensively to enhance bone health and mitigate the risk of osteoporosis (79, 80). Existing studies have shown that increasing milk intake in Asians may be beneficial to bone health (Figure 3; Table 4) (81). Vitamin D, a lipid-soluble vitamin, serves as a vital component of the “calcium-vitamin D-parathyroid hormone” endocrine axis, which is instrumental in maintaining calcium balance within the body (82). The bioactive form of vitamin D, known as 1,25-dihydroxy vitamin D, is essential for the regulation of both calcium and phosphate metabolism in the body (83). Vitamin D plays a pivotal role in facilitating the uptake of calcium in the small intestine, thereby aiding in the regulation of serum calcium concentrations. Active forms of vitamin D enhance the uptake of calcium from the intestinal tract, thereby improving the bioavailability of this essential mineral for bodily functions (84, 85). Similar to the previous text, vitamin D deficiency can also stimulate the secretion of parathyroid hormone, which in the long run can lead to bone loss and osteoporosis. Vitamin D supplementation is regarded as advantageous for both the prevention and management of osteoporosis. Another benefit of vitamin D for bones is that it can support collagen matrix and osteoblast mineralization, thereby promoting bone growth (86).

**Table 4:** Researches evaluating links between cow’s milk intake and bone mineral density.

Author, year	Journal	Study population	Study type	Result
Jae Hyun Lee (81), 2021	Nutrients	1,061 Korean people	A retrospective cross-sectional study	These results show that there is a synergistic effect of physical activity and milk intake on BMD, suggesting that practicing both moderate to vigorous physical activity and milk consumption in adolescence is an effective way to build healthy bones.
A Kojima (94), 2023	The Journal of Nutrition	1,429 postmenopausal Japanese women	A prospective cohort study.	High habitual milk intake is associated with a decreased risk of osteoporotic fractures, independent of bone mineral density, in postmenopausal Japanese women.
J-C Gui (93), 2012	Osteoporosis International	141 postmenopausal Chinese women and without osteoporosis	Randomized Controlled Trial	Daily consumption of milk containing 250 mg calcium over 18 months prevents BMD loss at the hip and the femoral neck in postmenopausal Chinese women
Ji Soo Kim (147), 2020	Korean Journal of Family Medicine	8,539 Korean people	A retrospective cross-sectional study	This study suggests that frequent milk consumption could potentially reduce osteoporosis incidence in South Korean adults.
Y Sato (92), 2015	Osteoporosis International	1,479 elderly Japanese men.	A prospective cohort study.	Greater milk intake was associated with lower bone turnover, higher BMD, and higher Trabecular Bone Score in community-dwelling elderly Japanese men
Shivani Shani (148), 2013	Archives of Osteoporosis	3,212 people	A prospective cohort study	Milk and yogurt intakes were positively associated with hip but not spine BMD
S Shin (149), 2015	European Journal of Clinical Nutrition	1828 Korean people	A cohort study	A dietary pattern with high intake of dairy products, fruits and whole grains may contribute positively to bone health in a Korean adult population, and dietary pattern-based strategies could have potential in promoting bone health.

The primary dietary source of vitamin D is food that has been fortified, particularly fortified milk, which is commonly enriched with this essential nutrient (45, 46). However, it should be noted that unreinforced milk has extremely low levels of vitamin D, and natural milk is hardly an effective dietary source of vitamin D. As few foods naturally contain vitamin D, fortified milk is a recommended pathway for vitamin D intake in many countries, and consuming fortified milk is an important food source for vitamin D supplementation (87, 88). Milk is naturally rich in calcium, and when fortified with vitamin D, its efficacy in promoting calcium absorption and utilization is maximized—an aspect that is particularly crucial for individuals at high risk of osteoporosis. In many regions across Asia, vitamin D deficiency is widespread due to factors such as limited sun exposure, dietary habits, and urban lifestyles (33, 34). Supplementing the diet with vitamin D–fortified milk, typically containing 400–1,000 IU/L, can help address this deficiency. This not only enhances calcium bioavailability but also supports optimal bone mineralization and health. For populations with a high prevalence of osteoporosis and vitamin D deficiency, regular consumption of vitamin D–fortified milk represents an effective and practical nutritional strategy to reduce fracture risk and improve overall bone health.

### 5.4 Impact of vitamin K2 on bone health

Vitamin K2 (particularly in the MK-4 and MK-7 forms) acts as a coenzyme in the *γ*-glutamyl carboxylation of vitamin K-dependent proteins, such as osteocalcin and matrix Gla protein (MGP), enabling these proteins to perform their biological functions. Vitamin K2 can also facilitate proper calcium deposition in bones. Activated osteocalcin effectively transports calcium and deposits it into the bone matrix, while MGP inhibits abnormal calcium deposition in blood vessels and soft tissues, reducing vascular calcification and enhancing bone mineralization efficiency (89, 90). Moreover, Vitamin K2 reduces fracture risk. Numerous clinical and epidemiological studies have shown that vitamin K2 supplementation increases BMD and significantly reduces the incidence of vertebral and non-vertebral fractures in postmenopausal women (91).

### 5.5 Associations between milk consumption and bone health

Numerous observational and interventional studies have investigated the relationship between milk consumption and various indicators of bone health, such as BMD, bone microarchitecture, and the risk of osteoporotic fractures. Milk is a rich source of calcium, high-quality protein, and other micronutrients essential for bone metabolism, including vitamin D (when fortified), calcium, and magnesium. Regular milk intake has been associated with higher BMD and improved bone strength in diverse populations, particularly among older adults and postmenopausal women, who are at increased risk for osteoporosis. Furthermore, some prospective cohort studies suggest that habitual milk consumption may contribute to a reduced risk of osteoporotic fractures. However, the magnitude and consistency of these associations can vary depending on factors such as age, sex, baseline dietary patterns, and genetic predispositions. Randomized controlled trials have provided additional evidence supporting the beneficial effects of milk and dairy intake on bone health outcomes (Table 4).

In a cohort of 1,479 community-dwelling older Japanese men (mean age 73.0 ± 5.1 years) with a median milk intake of one cup per day, higher milk consumption was associated with lower bone turnover rates, increased BMD, and improved bone microarchitecture indices. BMD was assessed using dual-energy X-ray absorptiometry (DXA), trabecular bone scores were measured at the lumbar spine using DXA images, and bone turnover was evaluated through biochemical markers in serum (92). Moreover, a randomized controlled trial conducted in China enrolled 141 Chinese women aged 45–65 years who were postmenopausal for more than 2 years and had no diagnosis of osteoporosis. Participants were randomly assigned to one of three groups for an 18-month intervention: (A) daily intake of milk providing 250 mg of calcium, (B) daily intake of soy milk providing 250 mg of calcium, or (C) no milk or soy milk intake. BMD of the spine and hip was measured at baseline, 6, 12, and 18 months using dual-energy X-ray absorptiometry. The results demonstrated that daily consumption of milk containing 250 mg of calcium for 18 months effectively prevented bone loss at the hip and femoral neck in postmenopausal Chinese women (93). In addition, in a prospective cohort study of 1,429 postmenopausal Japanese women with a median follow-up of 15.1 years, higher habitual milk intake was associated with a reduced risk of osteoporotic fractures. Baseline milk consumption frequency was obtained via nurse-administered questionnaires, while intake of yogurt, cheese, and estimated calcium was assessed using a validated food frequency questionnaire (94). It is worth noting that in a randomized trial involving 63 women over the age of 55, participants received either high-calcium, vitamin D-fortified milk or a control beverage, with two servings per day for 12 weeks. The fortified milk intervention significantly improved vitamin D status and reduced bone turnover rates over the 12-week period in postmenopausal Chinese women (95).

A substantial body of evidence from observational and interventional studies suggests that regular milk intake is associated with higher bone mineral density (BMD), improved bone microarchitecture, and a reduced risk of osteoporotic fractures, particularly in older adults and postmenopausal women. Randomized controlled trials and cohort studies in Asian populations support these findings, demonstrating that habitual milk consumption helps prevent bone loss and may lower fracture risk. The beneficial effects are attributed to milk’s rich content of calcium, high-quality protein, and other key nutrients essential for bone health. In summary, current evidence indicates that moderate milk consumption is associated with improvements in bone mineral density among older adults, postmenopausal women, and other populations at elevated risk for osteoporosis, supporting its role as an appropriate dietary component for these groups.

## 6 Lactose intolerance and milk allergy in Asians

### 6.1 Milk allergy

Allergy to cow’s milk is one of the most frequently encountered food allergies, affecting approximately 1.8 to 7.5% of infants within their first year of life. But in the majority of cases (85–90%) milk allergy resolve. Due to the lack of diagnostic infrastructure, professional diagnostic technology, and insufficient attention of the population, the implementation of large-scale population research in Asia is facing challenges. However, there are also some literatures that reflect the differences between different Asian countries and regions. A study conducted in Singapore revealed that the prevalence of a convincing history of cow’s milk allergy (CMA) in Singapore was 0.51% (95% confidence interval [CI], 0.3–0.7) (96). According to a survey conducted in southern China, the prevalence of CMA was 2.69%. CMA infants also have a strong family history of food allergy and atopy (97). According to a survey conducted in India, about 2.71% of the subjects’ serum test results showed allergy to milk. It is worth mentioning that only about one-third of the confirmed patients have clinical complaints (98). An Israeli study on the prevalence of food allergy in young children showed that about 1% of children had CMA (99). A review of the literature indicates that the prevalence of milk allergy does not significantly differ among various Asian countries. Caseins (especially *α* α-caseins) and *β*-lactoglobulin are considered the primary allergenic proteins in milk in allergic mechanisms (100). People frequently conflate milk allergy and lactose intolerance, although these are distinct allergy is an immune system response to proteins found in milk, whereas lactose intolerance results from a deficiency in lactase, the enzyme required to digest lactose, and does not involve the immune system. Milk allergies can manifest through either immunoglobulin E (IgE) mediated or non-IgE mediated pathways. The most common type I hypersensitivity in Asian population is mediated by immunoglobulin E (IgE). Reactions mediated by IgE typically present themselves promptly, often immediately following the consumption of milk. IgE-mediated cow’s milk allergy is classified as a Type I hypersensitivity reaction, with symptoms typically manifesting within a few minutes to 1 to 2 hours after ingestion. In this allergic response, antibodies specific to milk proteins, known as IgE, bind to mast cells (101). Subsequent exposure to these proteins triggers the mast cells to undergo degranulation, leading to the release of mediators including histamine. This release results in a variety of symptoms such as urticaria; angioedema; tightening of the throat; respiratory symptoms including difficulty breathing, coughing, and wheezing; gastrointestinal symptoms like abdominal pain, vomiting, and diarrhea; and cardiovascular symptoms, which can include dizziness, blurred consciousness, and hypotension. It should be pointed out that milk allergy in Asian children tends to show gastrointestinal symptoms rather than skin or respiratory symptoms (102–104). In contrast, non-IgE mediated reactions are characterized by a more delayed onset, with symptoms potentially appearing up to 48 h post-exposure, yet these responses still engage the immune system (105).

### 6.2 Lactose intolerance

Approximately 57% of the global population, predominantly in Africa and Asia, is affected by lactose intolerance. This widespread prevalence underscores the significant genetic and geographical variations in the ability to digest lactose among different populations worldwide (106, 107). According to existing research, Southeast Asian countries have the highest lactose intolerance rate in the world, with the prevalence ranging from 85 to 98%. Due to the influence of nomadic culture in history, countries in Central Asia have high lactose tolerance, with the prevalence ranging from 40 to 70%. The incidence rate in South Asia is about 60–80%. In East Asia, developed countries such as Japan and South Korea have a relatively low lactose tolerance rate of 75 to 85%, while China has reported an incidence rate of 80 to 90%. The prevalence of lactose intolerance in Western Asia is different. Generally speaking, nomadic peoples are relatively low, and the population bordering Europe is relatively low (108, 109). Lactose is the predominant carbohydrate found in the milk of mammals, with very limited occurrences of this specific carbohydrate in other natural sources (110). Lactose intolerance is a condition characterized by the inability to adequately absorb lactose, leading to the manifestation of various gastrointestinal symptoms (111). The specific symptoms are as follows: the onset of gastrointestinal symptoms such as gas, bloating, abdominal cramps, and pain, which are occasionally accompanied by mushy to watery diarrhea, and sometimes nausea and vomiting, following the consumption of significant quantities of foods containing lactose. The typical symptom of lactose intolerance in Asian population is abdominal pain, abdominal distension and diarrhea after eating dairy products (112). The majority of individuals are born with the capability to digest lactose, a carbohydrate that is broken down into glucose and galactose by the enzyme lactase. This enzyme is situated in the brush border microvilli within the small intestine, where it facilitates the hydrolyzation of lactose into its component sugars (113, 114). This malabsorption is primarily due to a deficiency in the enzyme lactase, which is essential for the digestion of lactose, the sugar found in milk and dairy products. The prevalence of lactase non-persistence (LNP) is highest among Asian populations, particularly in East and Southeast Asia. This condition is primarily associated with the C/T-13910 polymorphism in the promoter region of the LCT gene, with the vast majority of Asians exhibiting the C/C genotype, which is indicative of lactase non-persistence (115).

## 7 Possible strategies for mitigating milk allergy and lactose intolerance

### 7.1 Strategies for mitigating milk allergy

Exclusive breastfeeding is universally endorsed for all infants during the first 4 to 6 months of life owing to the numerous beneficial effects associated with breast milk. Additionally, there is mounting evidence suggesting that breastfeeding may play a role in reducing the risk of developing food allergies. This protective effect is attributed to the presence of immunomodulatory components in breast milk that may help in early immune system development, potentially decreasing the likelihood of allergic responses to food antigens later in life (116). Within this framework, it has been proposed that the presence of allergen-specific immunoglobulin A (IgA) and immunoglobulin G (IgG) antibodies in human breast milk serve as protective elements. These antibodies are believed to enhance the passive immunity that is transferred to infants during breastfeeding, potentially decreasing their vulnerability to allergic reactions. These specific antibodies in breast milk are thought to positively influence the development of the infant’s immune system, thereby reducing the likelihood of developing food allergies (117). According to the reliable data source of the United Nations Children’s Fund (UNICEF), the rate of early initiation of breastfeeding ranges from 45 to 50% in Southeast Asia, 20 to 30% in East Asia, approximately 52% in Central Asia, and around 50% in South Asia. In contrast, reliable estimates for West Asia and North Africa are limited, but available data suggest that the coverage is generally below 40%. In Southeast Asia, increasing urbanization, early maternal return to the workforce, and aggressive marketing of infant formula have contributed to shorter durations of early breastfeeding. The challenges are even more pronounced in East Asia, where urbanization is more advanced and women tend to resume work earlier, further limiting the time available for early breastfeeding. In South Asia, a significant proportion of births occur at home, and the implementation of early breastfeeding practices is often inadequate. Variations in maternal education levels and regional disparities in development further hinder awareness and adherence to optimal early breastfeeding practices. Similarly, in West Asia, rising urbanization and female labor force participation, combined with shorter maternity leave, have negatively impacted the initiation and duration of early breastfeeding. South Asia can focus on strengthening rural health services and family health education, leveraging the positive guiding role of religion and traditional culture in breastfeeding, ensuring timely and continuous breastfeeding after childbirth, and correcting misconceptions in low education areas. Governments in highly urbanized regions such as East Asia, Southeast Asia, and West Asia can promote the extension of maternity leave and increase flexible work policies, strictly restrict and regulate the formula milk market, raise public and healthcare workers’ awareness of prioritizing breast milk, and create a convenient breastfeeding environment for working mothers.

Recent research found that the early introduction of formula based on cow’s milk has been correlated with a diminished risk of developing cow’s milk allergy (CMA). This association suggests that introducing formula based on cow’s milk moderately to infants at a younger age may contribute to a decreased incidence of allergic reactions to this specific allergen (102, 118, 119). Analysis of data from a large Japanese birth cohort comprising over 100,000 mother–child pairs, involving 80,408 children, demonstrated that regular intake of cow’s milk–based formula during the first 3 months of life was linked to a reduced risk of cow’s milk allergy at 6 and 12 months of age (adjusted relative risks of 0.42 [95% CI 0.30–0.57] and 0.44 [95% CI 0.38–0.51], respectively) (120). Likewise, A total of 51 patients with IgE-mediated CMA were compared to 102 matched controls (1:2 ratio) and 32 unmatched patients with IgE-mediated EA. Multivariable logistic regression analysis revealed that delayed introduction (initiated after 1 month of age) or irregular consumption of cow’s milk formula (less than once daily) was associated with an adjusted odds ratio of 23.74 (95% CI: 5.39–104.52) when comparing the CMA group to the control group, and 10.16 (95% CI: 2.48–41.64) when comparing the CMA group to the EA group (121). It should be noted that the very early introduction of cow’s milk in the first days of life followed by a period of avoidance is associated with increased risk of developing CMA, and introduction of cow’s milk protein in the first hours to days after birth followed by inconsistent incorporation into the diet is associated with increased risk of developing CMA (122). Research has additionally indicated that temporarily removing cow’s milk from the diet can elevate the risk of developing a cow’s milk allergy. These findings imply that continuous consumption of formula based on cow’s milk, following its initial introduction, may be crucial in preventing the onset of this allergy, as sustained exposure helps promote immune tolerance to cow’s milk proteins. In many Asian countries, this evidence suggests a potential strategy for allergy prevention. Encouraging the regular and sustained introduction of cow’s milk products—especially in populations with rising formula use and evolving infant feeding practices—could be considered as a measure to reduce the incidence of cow’s milk allergy in Asian regions. However, such recommendations should be carefully tailored to local cultural, nutritional, and healthcare contexts (123, 124).

### 7.2 Strategies for mitigating lactose intolerance

Despite the high estimated prevalence of lactose intolerance, a substantial number of individuals with lactose malabsorption do not exhibit clinical symptoms or report issues related to lactose intolerance. This discrepancy highlights the variability in how lactose malabsorption manifests clinically among different individuals (125). Similarly, a significant number of individuals perceived to suffer from lactose intolerance do not exhibit symptoms. This observation suggests that the diagnosis of lactose intolerance may not always correlate directly with the clinical presentation of symptoms (126). These observational studies indicate that, despite the high prevalence of lactose intolerance in Asian countries, evidence-based medical strategies may offer effective means of mitigation.

The primary treatment strategy for lactose intolerance consists of initially restricting the diet to exclude lactose-containing foods, which is then followed by a carefully monitored reintroduction of these foods into the diet. This approach allows individuals to assess their level of tolerance and gradually adjust their intake accordingly (127, 128). The recommended management approach for lactose intolerance involves adhering to a diet that limits lactose intake. Research indicated that the overwhelming majority of individuals can tolerate up to 12 grams of lactose daily without experiencing significant symptoms, or with only minimal discomfort. However, establishing a definitive threshold for lactose intake is challenging due to the substantial variability in tolerance levels observed among different individuals (129). For example, we can gradually increase the intake of milk, starting from 30 to 60 mL per day and gradually increasing to a maximum of 250 mL per day. It is important to consume with meals rather than on an empty stomach to slow down the release of lactose in the small intestine (108, 115, 130). Considering the high incidence of lactose intolerance in the Asian population, some individuals may only be able to tolerate 250 mL of milk without causing clinical symptoms, regarding nutritional adequacy, while 250 mL of milk provides a substantial contribution to daily calcium and high-quality protein needs—for example, approximately 300 mg of calcium and 8 grams of protein—it represents only a portion of total daily requirements (108). Therefore, it is recommended that individuals complement milk intake with other calcium-rich foods (such as leafy greens, fortified products, or supplements) and maintain a balanced diet to achieve sufficient overall nutrient intake. Meanwhile, fermented dairy products and lactose free milk are also worth considering. Asian populations who are unable to tolerate regular milk may increase their intake of lactose-free milk and fermented dairy products. These alternatives can significantly alleviate symptoms of lactose intolerance commonly observed in Asian populations while providing comparable nutrients, such as calcium and protein. In addition, vitamin D-fortified milk is also recommended, as it contributes to improved nutritional status and helps improve vitamin D deficiency in Asian populations (112). Traditional diets in South Asia and West Asia predominantly feature fermented dairy products such as yogurt, lassi, and labneh, which naturally help alleviate symptoms of lactose intolerance. In contrast, in East and Southeast Asian countries—where the prevalence of lactose intolerance is particularly high—the promotion of lactose-free milk and fermented dairy products could serve as practical strategies. Additionally, governments should enhance public education on lactose intolerance, including distinguishing between lactose intolerance and milk allergy, through targeted health campaigns. Strengthening nutrition education can help consumers better understand the advantages and limitations of various dairy products and their alternatives. Furthermore, in light of widespread vitamin D insufficiency, the development and promotion of calcium- and vitamin D-fortified alternatives should be prioritized to ensure balanced nutrition. Lactase enzyme replacement is another option, but further studies are still required to support these strategies (131) (Figure 3).

## 8 Possible solutions to increase milk consumption and promote sustainable health in Asia

Possible strategies to enhance milk consumption and foster sustainable health in Asia must integrate multiple dimensions. Primarily, implementing comprehensive nutrition education and health promotion campaigns is essential to raise public awareness of milk’s nutritional benefits. By effectively disseminating scientific evidence on milk as a vital source of calcium and high-quality protein that supports bone development and overall health, consumer acceptance and motivation to consume milk can be significantly improved. Additionally, prolonging the duration of breastfeeding warrants promotion, as its beneficial effects on infants’ early immune function and gut health are well established, playing a crucial role in nutrient absorption and the prevention of gastrointestinal diseases (132).

Product diversification should not only address lactose content but also involve nutritional fortification strategies. The enrichment of milk with micronutrients, such as vitamin D, targets prevalent deficiencies in Asian populations, thereby enhancing the micronutrient profile and overall value of dairy products These products mitigate digestive discomfort associated with lactose intolerance, widen the consumer base, and enhance the overall consumption experience (133). From an economic perspective, efforts must be directed toward optimizing dairy production and distribution systems to reduce costs and improve supply chain efficiencies, particularly in rural and underserved regions, thereby increasing milk availability and affordability. Policy interventions are equally vital; governments should incorporate milk and dairy products into public nutrition frameworks and school feeding programs to secure the nutritional needs of vulnerable groups and foster healthy dietary habits from early childhood. Furthermore, environmental sustainability considerations are imperative. The adoption of green dairy farming practices and resource recycling measures to reduce greenhouse gas emissions and water use in animal agriculture is necessary to align nutritional improvements with ecological preservation (134).

## 9 Conclusion—guidelines for the use of milk in preventing and managing osteoporosis in Asians

The rapid increase in the aging population across Asia has been accompanied by a rising prevalence of osteoporosis and fracture incidence. Calcium and vitamin D deficiencies, combined with an aging population, place Asian populations at increased risk of osteoporosis. Traditional Asian diets, which are predominantly based on rice and wheat products, are typically low in calcium, magnesium, and high-quality protein. Low dairy intake constitutes a significant environmental factor contributing to the high prevalence of osteoporosis. Milk uniquely provides all three key nutrients essential for bone health—calcium, magnesium, and high-quality protein—almost simultaneously. Multiple cohort studies in Asian populations have demonstrated that dairy consumption is associated with higher BMD and a reduced risk of osteoporosis. Milk is also relatively affordable, portable, and easy to distribute, making it a nutritionally comprehensive beverage for the Asian population (135–137). At the same time, some milk and dairy products in Asian markets are fortified with vitamin D, which further enhances calcium absorption and helps address the common problem of vitamin D deficiency in Asian populations, thereby reducing the risk of osteoporosis. However, due to factors such as high lactose intolerance rates, dietary habits, cultural differences, and economic development levels, Asians have lower intake of dairy products and calcium ions (138). Although lactose intolerance affects a proportion of Asians, most individuals can tolerate at least one serving of milk without clinical symptoms. For Asian individuals who are unable to tolerate milk, dairy products such as yogurt and lactose-reduced milk provide safe and effective alternatives that meet the nutritional needs of most of the population and help address issues related to lactose intolerance. Challenges such as milk allergies among Asian populations may be mitigated through early initiation of breastfeeding, the gradual introduction of milk-based formula during infancy, and the maintenance of regular dairy consumption throughout life. Despite that some studies have found that consuming milk increases the risk of fractures, stroke, cardiovascular disease, and all-cause mortality (139–142), the current mainstream academic view confirms that milk has many benefits for health, especially for Asians at risk of osteoporosis. Overall, current evidence supports the health benefits of milk consumption for Asians at risk of osteoporosis, the challenges encountered can also be mitigated through targeted interventions and appropriate strategies.
